# The Impact of Pregnancy in Patients with Thoracic Aortic Disease: Epidemiology, Risk Assessment, and Management Considerations

**DOI:** 10.14797/mdcvj.1371

**Published:** 2024-03-14

**Authors:** Valeria E. Duarte, Jessica N. Richardson, Michael N. Singh

**Affiliations:** 1 Houston Methodist DeBakey Heart & Vascular Center, Houston, Texas, US; 2 Boston Children’s Hospital, Harvard Medical School, Boston, Massachusetts, US; 3 Brigham and Women’s Hospital, Harvard Medical School, Boston, Massachusetts, US

**Keywords:** pregnancy, aortopathy, Marfan, cardiovascular genetics

## Abstract

Thoracic aortic disease (TAD) poses substantial risks during pregnancy, particularly for women with genetic conditions such as Marfan syndrome, Loeys-Dietz syndrome, and vascular Ehlers-Danlos syndrome. This review examines the epidemiology, risk assessment, and management of TAD in pregnancy. Preconception counseling is vital considering the hereditary nature of TAD and potential pregnancy-related complications. Genetic testing and imaging surveillance aid in risk assessment. Medical management, including beta-blockade and strict blood pressure control, is essential throughout pregnancy. Surgical interventions may be necessary in certain cases. A multidisciplinary approach involving cardiologists, obstetricians, cardiac surgeons, anesthesiologists, and other specialists with expertise in cardio-obstetrics is essential for optimal outcomes. Patient education and shared decision-making play vital roles in navigating the complexities of TAD in pregnancy and improving maternal and neonatal outcomes.

## Introduction

In one population study, the incidence of thoracic aortic disease (TAD) was observed to be 10.4 per 100,000 person-years. Remarkably, 51% of TAD cases were identified in women, who, on average, were considerably older at the time of diagnosis than men.^1^ Furthermore, women are more likely to present with imaging findings suggestive of rupture, have in-hospital complications, and have higher surgical (32% vs 22% in type A dissection) and in-hospital mortality compared with men.^2^

Thoracic aortic aneurysms are associated with genetic syndromes such as Marfan syndrome (MS), Loeys-Dietz syndrome (LDS), and vascular Ehlers-Danlos syndrome (EDS) as well as Turner syndrome, familial thoracic aneurysm, bicuspid aortic valve (BAV), and coarctation of the aorta. Women with these conditions are at risk for pregnancy-related aortic dilation and dissection, representing one of the main causes of cardiovascular death during pregnancy.^3,4,5^

The physiological hemodynamic changes of pregnancy—including increased blood volume, heart rate, and stroke volume, decrease of systemic vascular resistance, and hormonal changes of pregnancy—increase the risk of progressive aortic dilation or dissection in these women both during pregnancy and in the long term.^6,7,8,9,10,11^ Histologic analysis of arterial tissue samples of postpartum women who suffered aortic dissection demonstrated significant arterial degeneration and loss of integrity.^12^

This review explores risk assessment, counseling, and management strategies for thoracic aortic disease before and during pregnancy and in the postpartum period. TAD also is associated with other congenital conditions that exceed the scope of this document.

## Epidemiology

Aortic dissection represents an uncommon yet life-threatening complication that can occur during pregnancy.^13,14,15^ Most of these events surface in the third trimester or in the first 12-week postpartum period, but it can occur earlier in pregnancy and may occur after prior aortic root replacement.^15^ A large prospective global registry, the Registry of Pregnancy and Cardiac Disease (ROPAC), which included nearly 6,000 pregnant women with cardiovascular disease, reported thoracic aortic disease in 189 (3.3%) of the enrolled patients (58% with aortic dilation and 6% with a prior history of aortic dissection); half of them carried a diagnosis of MS, 26% of BAV, 8% of Turner syndrome, 2% of vascular EDS, and 11% had no underlying genetic defect or associated congenital heart defect. In this analysis, there were four cases of dissection (three type A, one type B), three of which occurred in women with MS. There was no maternal or fetal mortality.^3^

In a cohort study of the International Registry of Acute Aortic Dissection, pregnancy-related aortic dissections represented 0.3% of all dissections and 1% of aortic dissection in women. The majority of these occurrences were observed during the third trimester and postpartum period. Among affected individuals, 69% had a prior diagnosis of aortopathy or positive family history, 65% had MS, 10% had LDS, and 10% had a bicuspid aortic valve. Notably, aortopathy was not recognized until after the occurrence of aortic dissection in 47% of the affected women. The survival rate following aortic dissection was 97%.^15^ Although occurring infrequently, in other studies aortic dissections contributed even more significantly to maternal mortality. In a prospective nationwide cohort study covering all deliveries in the Netherlands over a 2-year period, the maternal mortality rate was 3 per 100,000 deliveries. Remarkably, 45% of these deaths were attributed to aortic dissection.^13^

An analysis of 75 cases of acute aortic dissection during pregnancy documented in the literature revealed that 77% of the cases were classified as Stanford type A dissections. The majority (78%) of these occurred during the third trimester and the immediate postpartum period. Underlying connective tissue disorders were present in 49% of the patients. The mortality rate was recorded at 22%. In cases necessitating antepartum repair, fetal mortality stood at 36%.^16^

Marfan syndrome has an important association with complications in the available databases. In a retrospective observational study of women with MS delivering over a span of 20 years in 12 UK centers, 258 pregnancies in 151 women with MS were analyzed. Notably, only 50% of the women received preconception counseling. There were no deaths, but five women (1.9%) experienced aortic dissections (one type A and four type B), and five women required cardiac surgery postpartum. The Cesarean section (C-section) rate was high at 50%, especially in women with dilated roots. There was a 0.84-mm increase in aortic root size.^17^ The National Registry of Genetically Triggered Thoracic Aortic Aneurysms and Cardiovascular conditions (GenTAC) reported outcomes of 184 women with MS who participated: 10 (10.6%) experienced a pregnancy-related aortic complication (four type A and three type B dissections, one coronary artery dissection, and two significant [≥ 3 mm] aortic growth). Five of the seven aortic dissections and the coronary dissection occurred in the postpartum period. Only five of eight women with pregnancy-associated dissection were aware of their MS diagnosis.^4^

Beyond the immediate impact of pregnancy, one analysis of 69 women with MS notably did not have dissection or require surgery during pregnancy, although they had increased obstetric complications (10%) and adverse fetal outcomes (13%). In addition, on long-term follow-up, aortic growth rate increased during pregnancy and did not return to baseline after pregnancy, and the prevalence of aortic dissection and elective aortic surgery was higher for those women who had a prior pregnancy.^10^

Loeys-Dietz syndrome predisposes patients to particularly aggressive and widespread vascular disease that results in elevated mortality rates at a young age. A landmark study involving 90 patients with LDS type I and type II from 52 affected families outlined a natural history marked by the development of aggressive arterial aneurysms (with a mean age at death of 26 years) and a considerable incidence of pregnancy-related complications, affecting 6 out of 12 women (aortic dissection in four and uterine rupture in two).^18^ There is very limited data about pregnancy outcomes in women with LDS who have undergone prior aortic root replacement. In a case series, two-thirds of women with LDS who had undergone prophylactic root replacement suffered acute dissection in the postpartum period.^19^

There is a paucity of data on pregnancy-related risk and outcomes in patients with vascular EDS. In a review of pedigrees and interviews of families with vascular EDS, pregnancy-related death occurred in 30 of 565 deliveries (5.3%). In a subgroup analysis of 76 of these pregnancies (in 35 women who were interviewed for details), 42.2% of the patients underwent C-sections (4 of 25 were emergent). The most common pregnancy-related complications were third-/fourth-degree lacerations (20%) and preterm delivery (19%). Life-threatening complications occurred in 14.5% of deliveries and included arterial dissection/rupture (9.2%), uterine rupture (2.6%), and surgical complications (2.6%). There were five maternal deaths (6.5%), resulting from arterial dissection or rupture in four patients, and complications related to dehiscence of a C-section incision in one.^17^

Spontaneous pregnancy can occur in patients with mosaic Turner syndrome (0.5-10% incidence), but pregnancy in women with Turner syndrome most commonly results from assisted fertility methods. Comprehensive cardiovascular evaluation and aortic imaging should be performed prior to initiation of fertility treatments, as dissection during pregnancy can occur and has been reported in pregnancy resulting from assisted reproduction technologies.^20^

Limited data are available regarding non-syndromic hereditary thoracic aortic disease (nsHTAD) during pregnancy; however, it is noteworthy that aortic dissections have been documented in these patients, even at smaller aortic diameters. In a study involving 53 women with a total of 137 pregnancies, eight experienced aortic dissections during the third trimester or postpartum period (6% of pregnancies). Among these, six dissections originated in the ascending aorta (Stanford type A), with three resulting in fatalities. Importantly, three women experienced ascending aortic dissections with diameters less than 5.0 cm (ranging from 3.8 to 4.7 cm).^21,22^

Aortic dissection related to BAV is rare and occurs in patients with associated aortic dilation.^3,15^ Due to the infrequency of this condition and its diverse manifestations, diagnosis may be overlooked. Maintaining a high level of suspicion when a woman presents with symptoms suggestive of the condition and making a prompt diagnosis could enhance maternal and neonatal outcomes.^14^

## Pre-Pregnancy Care

### Genetic Considerations and Counseling

Approximately 20% to 25% of individuals with thoracic aortic disease possess an underlying pathogenic variant consistent with Mendelian inheritance. These conditions are collectively termed heritable thoracic aortic diseases (HTAD), representing a group of disorders that are both clinically and genetically diverse.^5,23^ The 2022 Aortic Disease Guidelines recommends genetic counseling to discuss the heritable nature of their condition before pregnancy in patients with genetic arthropathies due to HTAD.^5^ Eleven genes have been identified as conferring a highly penetrant risk for HTAD: *FBN1, LOX, COL3A1, TGFBR1, TGFBR2, SMAD3, TGFB2, ACTA2, MYH11, MYLK*, and *PRKG1*.^5,23^ Regular discoveries of new genes underscore the rapid advancement of this field and reveal existing gaps in knowledge.

HTAD can be classified into two primary categories: syndromic and non-syndromic. Syndromic HTAD is linked to genetic syndromes that involve multiple systems, with MS being the most prevalent, followed by other less-common connective tissue syndromes such as LDS and vascular EDS. Conversely, non-syndromic HTAD refers to a cohort of individuals who harbor genetic mutations impacting the aorta and possibly its branches, albeit without affecting other systems (without recognizable phenotypes) and are associated to pathogenic variants in multiple genes affecting extracellular matrix proteins, transforming growth factor beta signaling, and smooth muscle contractile function. The pattern of inheritance of these conditions is autosomal dominant, with an inheritance risk of 50%.^24^ Additionally, there exists a subset of patients who, despite having a familial history of TAD, either possess genetic mutations of undetermined pathological significance or have had no detected mutations.^25,26,27^ In 25% of families exhibiting distinct signs of a familial thoracic aortic aneurysm (TAA) or dissection condition, a genetic mutation is detected in one of the recognized HTAD genes. In the absence of a genetic diagnosis and in cases where familial TAA is suspected, screening for first-degree relatives should commence at 25 years of age or 10 years prior to the age of onset in the youngest affected individual within the family.^24,28^

The presence of a BAV frequently leads to enlargement of the ascending aorta, and the aortic root in a smaller subgroup. Anomalous blood flow patterns associated with BAV and/or genetic abnormalities may contribute to aortic enlargement. BAV and ascending thoracic aortic disease can have a hereditary component, following an autosomal dominant inheritance pattern characterized by variable expression and incomplete penetrance. Although numerous genetic variations have been detected in individuals and families with BAV and TAA (including *TGFBR1, TGFBR2, TGFB2, TGFB3, ACTA2, MAT2A, GATA5, SMAD6, LOX, ROBO4*, and *TBX20*), most patients do not have an identifiable genetic variant linked to the condition. BAV can coincide with familial TAA conditions, and there is an increased incidence of BAV in individuals with LDS and certain non-syndromic hereditary thoracic aortic diseases. Consideration of genetic testing for HTAD may be warranted in BAV patients presenting with early-onset TAA, familial TAA, aortic root aneurysms, or syndromic characteristics.^24,29,30^

### Preconception Counseling

The discussion of pregnancy risks and implications should be integrated into routine cardiovascular assessments for women of childbearing age with arthropathies. In addition, women who are at risk for aortopathy due to phenotypic features of syndromes associated with HTAD and/or a family history of aortopathies should be referred to a cardiovascular center of expertise for comprehensive evaluation, including imaging and genetic testing, particularly if the affected first-degree relative has known pathogenic genetic variants.^31,32^

The importance of timely prepregnancy counseling for all women with established aortic conditions who intend to conceive cannot be overemphasized. Various considerations necessitate discussion encompassing long-term prognosis, the hereditary risk associated with each condition, pharmacological interventions risks and benefits, the risks of aortic dissection related to pregnancy, maternal risk estimation and outcome, anticipated fetal outcomes, and individualized plans for pregnancy management and delivery.^5,33^ Informed shared decision-making is crucial.^5,33^ If pregnancy is not desired, patients should be referred to a specialist to discuss reliable contraception options.

### Preconception Risk Assessment

In patients with established aortopathy, aortic imaging with echocardiography, cross-sectional imaging of the entire aorta (with cardiac magnetic resonance or computed tomography), or both should be performed before pregnancy to determine aortic dimensions for risk stratification. The risk of type A dissection during pregnancy is correlated with the underlying aortopathy and aortic diameter. However, type B aortic dissection may occur even in the absence of substantial aortic dilation.^4,5,15^ It is important to consider body surface area (BSA) in women of small stature and Turner syndrome. In MS, type A aortic dissection risk is very low when aortic diameters are < 4 cm and are much higher at diameters > 4.5 cm.^5^

The modified World Health Organization (mWHO) classification is presently regarded as the most precise system for risk assessment. However, its applicability may be more suitable for developed nations rather than those in the developing world.^33,34,35^ According to the mWHO, Turner Syndrome without aortic dilation is considered risk II (small increased risk of maternal mortality or moderate increase in morbidity, maternal cardiac event rate [MCER] of 5.7-10.5%). The following scenarios are considered mWHO category II-III (intermediate increased risk of maternal mortality or moderate to severe increase in morbidity, MCER: 10-19%): MS or other HTAD syndrome without aortic dilation, aorta < 45 mm in BAV pathology, and repaired coarctation. The following scenarios are considered mWHO III (significantly increased risk of maternal mortality or severe morbidity, MCER: 19-27%): moderate aortic dilations (40-45 mm in MS or other HTAD; 45-50 mm in BAV, Turner syndrome with aortic sinuses of Valsalva index [ASI] 20-25 mm/m^2^ BSA).

### Contraindications for Pregnancy

The mWHO classification of maternal cardiovascular risk places the following clinical scenarios in category IV (maternal cardiac event rate 40-100%, with extremely high risk of maternal mortality or severe morbidity; pregnancy is contraindicated): individuals with severe aortic dilation, including those with HTAD such as Marfan syndrome (aortic diameter > 45 mm), BAV (aortic diameter > 50 mm or > 27 mm/m^2^ BSA), or Turner syndrome (ASI > 25 mm/m^2^ BSA).^33^ The 2018 ESC Guidelines for the management of cardiovascular disease during pregnancy recommends against pregnancy in any patients with a history of aortic dissection, patients with LDS and thoracic aorta > 45 mm (or > 40 mm with family history of dissection or sudden death), and patients with vascular EDS.

### Pre-Pregnancy Surgical Intervention

The decision to pursue surgical intervention on the thoracic aorta in a woman considering pregnancy is intricate and contingent upon the particular disorder, genetic mutation, rate of aortic expansion, familial history, and phenotype. The potential risks associated with aortic surgery should be carefully evaluated. While prophylactic aneurysm surgery can mitigate the risk of proximal aortic dissection, there remains a possibility of pregnancy-related dissection occurring distal to the aortic graft in individuals with HTAD.^5^ This risk may be elevated, particularly in women with LDS.^18^ The 2022 American College of Cardiology (ACC)/American Heart Association (AHA) Guidelines for the Diagnosis and Management of Aortic Disease recommend that aortic surgery prior to pregnancy be considered in patients with nsHTAD and aortic diameter ≥ 4.5; if the aortic diameter is 4.0 cm to 4.4 cm, surgery can be considered depending on the genetic diagnosis, family history, and aortic growth rate. In addition, surgery should be considered in patients with Turner syndrome and ASI of ≥ 2.5 cm/m^2^, in patients with BAV (in the absence of TS or other HTAD) and aortic diameter ≥ 5 cm, and in patients with sporadic aortic root aneurysms, ascending aortic aneurysms, or both and a diameter of ≥ 5 cm.^5^

## Pregnancy Care

### Multidisciplinary Approach

In patients with aortic aneurysms or at increased risk of aortic dissection, pregnancy should be monitored by a multidisciplinary team at a center where an emergency aortic repair can be performed.^5^ Tertiary centers have recently focused on building comprehensive cardio-obstetrics teams to care for pregnant patients with cardiovascular disease.^6^ The essential team members should include a cardiologist, obstetrician, and anesthesiologist, all possessing specialized knowledge in managing high-risk pregnancies in women with heart disease. Depending on the specific circumstances, additional specialists may be required, such as a geneticist, cardiothoracic surgeon, pediatric cardiologist, fetal medicine specialist, neonatologist, hematologist, nurse specialist, pulmonary specialist, and other relevant experts.^33^ Patients with aortopathies have better outcomes when managed by a cardiovascular team with expertise.^10^ Specifically, women with a moderate or high risk of complications during pregnancy (mWHO Categories II-III, III, and IV) should be managed at expert centers for pregnancy and cardiac disease. A comprehensive interdisciplinary management strategy should be formulated and communicated to the patient, and this plan should be available in the patient’s chart.^33^

It is essential to educate patients and family members on the signs and symptoms of aortic dissection as well as a plan of action since they may help with timely presentation and management to improve outcomes.^5,14^

### Imaging Surveillance

All individuals with aortopathy and aneurysms involving the aortic root and ascending aorta should undergo regular surveillance imaging using transthoracic echocardiography (TEE) during each trimester of pregnancy and in the postpartum period. The frequency of imaging may be increased based on factors such as aortic dimensions, growth risk, and underlying medical conditions. For patients with aneurysms affecting the aortic arch, descending, or abdominal aorta, cardiac magnetic resonance imaging (CMR) without gadolinium is recommended over computed tomography to minimize repeated radiation exposure.^5,33^ It might be preferred to avoid CMR in the first trimester based on limited data. The use of gadolinium-based contrast agents during pregnancy remains controversial due to concerns about potential fetal effects, as gadolinium is water soluble and capable of crossing the placenta into the fetal circulation and amniotic fluid.^36,37^ If clinically indicated and likely to impact clinical decision-making and management, computed tomography angiography with iodine contrast may be performed.^38^ A TEE can be performed safely during pregnancy if required or if other modalities are not available or feasible.^5^

### Medical Management

The Aortic Disease Guidelines recommend beta-blocker therapy for pregnant and postpartum patients with aortopathy unless contraindicated.^5^ Fetal growth rate has been reported to be affected. However, data from the ROPAC registry is reassuring.^3^

Hypertensive disorders of pregnancy should be managed aggressively, adhering to guideline recommendations that include vigilant and frequent blood pressure monitoring as well as timely adjustments in medication dosages.^6,33,39^ Strict diabetes and hypertension surveillance and management are particularly important in patients with Turner syndrome during pregnancy.

In countries where it is commercially available, celiprolol is recommended for individuals diagnosed with vascular EDS, even in normotensive women, given their high risk and because a small randomized, multicenter, open trial demonstrated significant benefits in decreasing the rate of dissection and rupture.^40^ Celiprolol is currently not available in the United States. Type B dissection should be managed medically with medications that are safe during pregnancy.^33^

### Surgical Intervention During Pregnancy

Prophylactic surgery should be considered during pregnancy if the aorta diameter is > 45 mm and increasing rapidly. If the fetus is viable, C-section followed by aortic surgery is recommended.

In the event of type A aortic dissection, emergent C-section followed by aortic surgery should be performed if there is fetal viability. In cases of early pregnancy when the fetus is still not viable, aortic surgical repair with continuous fetal monitoring should be performed. It should be noted that even though the maternal outcome is good, fetal mortality is high (20-30%). In a report of 11 pregnant women undergoing aortic surgery, there were no maternal deaths, but three pregnancies resulted in intrauterine demise within a week of surgery. The mean cardiopulmonary bypass and cross-clamp time in the study were 105 and 89 minutes.^41^

### Labor and Delivery Considerations

Labor induces hemodynamic and hormonal shifts, heightening the risk of aortic dissection. Delivery for all pregnant women with aortic dilation or a history of aortic dissection is advised to take place at an experienced center equipped with a cardio-obstetrics team and access to cardiothoracic surgery.^33^ Beta-blockers should be continued in the peripartum period. In patients with an aortic root or ascending aorta diameter < 4 cm, vaginal delivery is recommended. In patients with a thoracic aorta measuring 4 to 4.5 cm, vaginal delivery with epidural anesthesia and assisted second stage should be considered.^30,42^ Patients who should be considered for C-section include patients with an aortic root or ascending aorta diameter > 4.5 cm and patients with a history of dissection.^33^ In addition, the 2022 ACC/AHA Guidelines for the Diagnosis and Management of Aortic Disease recommend considering C-section in patients with syndromic and ns-HTAD and a diameter of the aortic root or ascending aorta of 4 to 4.5 cm.

When feasible, it is not advisable to use ergometrine in women with aortic disease.^33,43^

## Conclusions

Our review highlights the complexities surrounding the diagnosis, management, and outcomes of thoracic aortic disease in pregnancy. Despite being an infrequent complication of pregnancy, aortic dissection represents a significant risk to maternal and fetal health, necessitating a multidisciplinary approach involving cardiologists, obstetricians, anesthesiologists, cardiac surgeons, and other specialists. The identification of high-risk individuals, such as those with hereditary thoracic aortic disease, underscores the importance of preconception counseling and close monitoring throughout pregnancy. Strict blood pressure control and beta blockade are the cornerstones of medical management. Because of the rarity and diverse presentation, diagnosis can be overlooked. Vigilance when a woman presents with symptoms suggestive of dissection could lead to a timely diagnosis, potentially improving maternal and neonatal outcomes. Education of the patient and family members on the signs and symptoms of aortic dissection is essential. Management should be tailored to the unique features of each individual, and surgery before and during pregnancy requires a multidisciplinary discussion, as does the prioritization of informed shared decision-making with the patient.

## Key Points

- Thoracic aortic disease (TAD) poses substantial risks during pregnancy due to the potential of hereditary pregnancy-related complications.
- For women with TAD, genetic testing and imaging surveillance can aid in risk assessment, and preconception counseling is vital.
- Medical management, including beta-blockade and strict blood pressure control, is essential throughout pregnancy for women with TAD. Surgical interventions may be necessary in certain cases.
- A multidisciplinary approach to TAD in pregnancy will involve cardiologists, obstetricians, cardiac surgeons, anesthesiologists, and other specialists in cardio-obstetrics. Patient education and shared decision-making are vital for improving maternal and neonatal outcomes.
